# *Fructus Mume* Formula in the Treatment of Type 2 Diabetes Mellitus: A Randomized Controlled Pilot Trial

**DOI:** 10.1155/2013/787459

**Published:** 2013-03-07

**Authors:** Xiang Tu, ChunGuang Xie, Fei Wang, Qiu Chen, ZhiHuang Zuo, Qiong Zhang, XingShuan Wang, Sen Zhong, James B. Jordan

**Affiliations:** ^1^National Traditional Chinese Medicine Clinical Research Base for Diabetes Mellitus, Teaching Hospital of Chengdu, University of Traditional Chinese Medicine, Chengdu, Sichuan 610072, China; ^2^Department of Endocrinology, Teaching Hospital of Chengdu, University of Traditional Chinese Medicine, Chengdu, Sichuan 610072, China; ^3^College of Clinical Medicine, Chengdu University of Traditional Chinese Medicine, Chengdu, Sichuan 610075, China; ^4^Central Hospital of Guangyuan City, Guangyuan, Sichuan 628000, China; ^5^Hospital (T.C.M.) Affiliated to Luzhou Medical College, Sichuan 646000, China; ^6^Traditional Chinese Medicine Hospital of Suining City, Sichuan 629000, China; ^7^Southwestern College, 3960 San Felipe Roud Santa Fe, NM 87507, USA

## Abstract

*Background*. “*Fructus Mume* or Dark Plum” (pilule form) has been used for many years in Traditional Chinese Medicine (TCM) and may be a valid treatment for type 2 diabetes mellitus (T2DM). *Aim*. One aspect toward efficacy validation is the evaluation of the blood glucose-lowering effect of *Fructus Mume* (*FM*) with T2DM patients in a randomized controlled trial (RCT). *Methods*. This pilot study uses a RCT procedure to assess efficacy of *FM* and Metformin. The trial was for 12 weeks, with 80 T2DM subjects. Both groups were standardized in their diet and exercise routine. Comparisons of several variables were analyzed. *Results*. No significant differences were found between groups in the fasting and postprandial glucose levels although both had significant decreases. The values of glycosylated hemoglobin were significantly reduced in both groups. For patients whose body mass index (BMI) was <23, neither *FM* nor Metformin had an effect on BMI; for those with a BMI between 23 and 25 or the BMI was >25, both *FM* and Metformin significantly reduce the BMI. *Conclusions*. In this pilot study, it was demonstrated that *Fructus Mume* formula may reduce the levels of blood glucose in patients with type 2 diabetes.

## 1. Introduction

It has been said that some traditional chinese medicinal (TCM) formulae could provide clinical benefits for patients with type 2 diabetes mellitus [1]. The reported benefits include optimal glycemic control, amelioration of clinical manifestations, and preventing or improving macrovascular or microvascular complications.

A TCM formula called “*Fructus Mume*” (Chinese: “Wumei Wan,” Dark Plum fruit pilule) is used to lower blood glucose levels. It was first recorded in the *Treatise on Cold Damage Diseases* (Chinese: “Shanghanlun”) which was written by the Sage of TCM-Zhang ZhongJing (circa 200–205 CE). Two animal experimental studies declared that it has blood glucose-lowering effect by facilitating the recovery of islet *β*-cells, increasing the concentration of hepatic glycogen, accelerating the glycogen synthesis, stimulating *β*-cells to excrete insulin, improving the glucose utility of peripheral tissues, and so forth [2].

The present researchers assert that the *FM* formula (composed of ten herbs; Table 1) has potential in the treatment of diabetes. First, it contains some special herbs which may be directly effective for diabetes. Among these herbs, *coptidis* is a herb of bitter flavor and cold property and *Dark Plum* is of sour flavor. *Four-qi* and *Five flavors* represent the main effects of Chinese medicinals and are one of the basic concepts in TCM theory. It is therefore necessary in our research to explain the hypoglycemic effect of *FM* besides *Yin-Yang *theory. There is no direct correlation of the use of TCM concept vocabulary such as *flavors* (sour, bitter, and sweet) with Western scientific concepts, so we are limited in our explanation of these concepts. In TCM theory, bitter flavor is in direct opposition to sweet flavor, and sour flavor can neutralize sweet flavor [3]. So the combination of bitter and sour flavors is an excellent approach to counteract sweet flavor. Secondly, it incorporates many basic TCM principles into a formula by using herbs of various flavor and properties [4]. Both hot and cold properties are also in the *FM* formula. This reflects the basic concept of *Yin-Yang* in TCM theory, and we interpret this formula as a typical prescription where herbs of cold or hot property are used together to adjust the balance of *Yin-Yang*. Generally speaking, it is a formula highly revered by ancient and modern TCM practitioners.

The individual substance alone in the herbs used in *FM* may have beneficial effects on diabetes. For example, initial research with berberine and ginsenosides is promising.

*Berberine. Coptidis* is one of the most popular herbs for diabetes [5], as it contains *berberine*. Numerous studies have demonstrated that *berberine* could exert beneficial effects on the treatment of diabetes [6]. The potential mechanisms include improving insulin sensitivity, inhibiting gluconeogenesis, stimulating glucose uptake through the AMP-AMPK-p38 MAPK pathway, or correcting lipid disorders [7–11]. Some articles have examined the effects and safety of *berberine* among patients with type 2 diabetes and suggested that it is effective and safe [12–14].

*Ginsenosides.* A recent article [15] has declared that malonyl ginsenosides (one of the natural ginsenosides of ginseng, another herb used in *FM*) could alleviate hyperglycemia, hyperlipemia, and insulin resistance of type 2 diabetes. Cho and coworkers [16] reported that ginsenoside Re could lower blood glucose and lipid levels and exerts protective actions against the occurrence of oxidative stress in the eye and kidney of diabetic rats. 

But all of these researches have not provided evidence to demonstrate the blood glucose-lowering effect of *FM* on well-designed randomized controlled trials.

Although many benefits have been reported by the TCM community, there is one question that remains unanswered; are TCM methods able to reduce the levels of blood glucose? The current debate over the role of TCM in management of diabetes mellitus revolves around its ability to serve as an independent monotherapy versus being delegated to the role of adjunct to hypoglycemia agents. The present pilot study is a randomized controlled trial designed to evaluate the blood glucose-lowering effect and safety of the *Fructus Mume* formula in patients with type 2 diabetes mellitus. One of the ingredients of the *FM* formula, *Herba Asari, *(Manchurian Wild Ginger) contains aristolochic acid and is therefore nephrotoxic [17]; we therefore removed this herb from the formula. As *Rhizoma Typhonii Gigantei* remained in our test drug and plays a major role as an extreme hot herb in *FM *[18], removing *Herba Asari* will not upset the balance of hot/cold, yin/yang. The ingredient herbs of the modified *FM* are shown in Table 1. 

The present study was registered on the Chinese Clinical Trial Registry: ChiCTR-TRC-12002320. The results are presented according to the Consolidated Standards for Reporting Trials of Traditional Chinese Medicine (CONSORT for TCM) checklist [19].

## 2. Subjects and Methods

### 2.1. Subjects

The study was conducted at four centers in Sichuan Province, China. The ethics committee of the Teaching Hospital of Chengdu University of Traditional Chinese Medicine approved the protocol, and all patients provided the written informed consent. The diagnosis of type 2 diabetes was based on clinical history and the finding of blood glucose concentrations according to the China Guideline for Diabetes Prevention and Treatment. To be included in the study, all patients had to have 7.0 mmol/L (126 mg/dL) ≤ fasting plasma glucose (FPG) ≤ 13.3 mmol/L (240 mg/dL) or 11.1 mmol/L (200 mg/dL) ≤ 2 h postprandial plasma glucose (2hPG) ≤ 22.9 mmol/L (412 mg/dL). Other inclusion criteria included an age of 18 to 70 years and normal renal function, and the value of transaminase was lower than one and a half times the upper limit. Patients were excluded if they had any of the following: pregnancy or lactation, a history of alcohol abuse, a history of cerebrovascular accident, malignant hypertension and acute coronary syndrome within the previous six months, allergic constitution, or an allergic history to TCM. Patients were also excluded if they had taken medications which are known to affect glucose metabolism such as thiazide diuretic; nicotinic acid within the past three months; or had comorbidity such as chronic heart failure; chronic renal failure; and hematopoietic system disease, mental disorders; had diseases which are known to affect glucose metabolism such as thyroid disease and adrenal gland disease; had participated in other clinical trials within the past three months. 

The baseline characteristics of the two groups of patients are shown in Table 2. In this pilot study, there are more female subjects in the Metformin group. Other baseline characteristics did not vary greatly.

### 2.2. Interventions

A total of 85 subjects were randomized to receive either *FM* or Metformin. Metformin is commonly used for type 2 diabetes, and clinical trials demonstrate it is safe and efficacious [20] in reducing plasma glucose concentrations in patients with T2DM. All patients received diet and exercise therapy. The randomization sequence was generated with an SAS software package by the Good Clinical Practice (GCP) Center of the Teaching Hospital of Chengdu University of TCM. The sequence was concealed and disseminated using opaque envelopes. All patients were instructed with a diet therapy (developed by a national Chinese nutrition society) where the calculated daily calorie expenditure was given, and recommended daily dietary nutrients were provided. 

To insure control over the quality of the decoction, this paper was standardized between research centers. All Chinese medicinals were processed according to the “Pharmacopoeia of the People's Republic of China” (2010 edition) and purchased from Sichuan Neautus Traditional Chinese Medicine, Inc., Ltd. Before decoction, the herbs of *FM* were infused for half an hour. The *Giant Typhonium Rhizome* was decocted firstly for one hour and then the other herbs of *FM* were added, decocted for another half an hour under 0.1 MPa, 120°C. Herbs were decocted with an automatic boiling and packaging machine, using three packages of decoction (200 mL/package). Patients were orally administered three packages daily. Metformin was taken at the dose of 500 mg twice daily.

### 2.3. Analytic Methods

Plasma glucose was measured every four weeks with a Hitachi analyzer by the hexokinase (HK) method, and glycosylated hemoglobin was measured by high-performance liquid chromatography at randomization and week 12. Blood chemical tests were performed with a 7170A automatic analyzer (Johnson & Johnson Medical (China) Ltd.) and urinalysis was performed with an AVE-763B automatic analyzer at randomization and week 12. The levels of insulin were determined by the electrochemiluminescence (ECL) method with a Cobase 601 analyzer at randomization and week 12. 

### 2.4. Statistical Analyses

All analyses were performed on a modified intention-to-treat population. The full-analysis-set (FAS) population was the primary population for assessing efficacy. The FAS included patients who took at least one dose of the study drug and had at least one value on treatment. Missing data were imputed with the use of the last-observation-carried-forward method, whereby missing values were replaced by the last nonmissing value. A worst case scenario (WCS) was conducted for the proportion of patients with FPG < 7.0 mmol/L; namely, the patients who dropped out were assumed to have achieved FPG < 7.0 mmol/L in the Metformin group, and those in the *FM* group were assumed to have not. The Pearson Chi-Square test was used to determine the difference in the proportion of patients with FPG < 7.0 mmol/L or 2 h PG < 10.0 mmol/L between the two groups. Repeated measures and multivariate analysis of variance of the general linear model were applied to determine the changes in blood glucose levels. The changes in body mass index were summarized and subgroup analyses were made by *t*-test according to the Chinese body size, namely, BMI < 23 indicating normal, BMI 23 to 25 indicating overweight, and BMI > 25 indicating obesity.

## 3. Results

**Figure 1:**
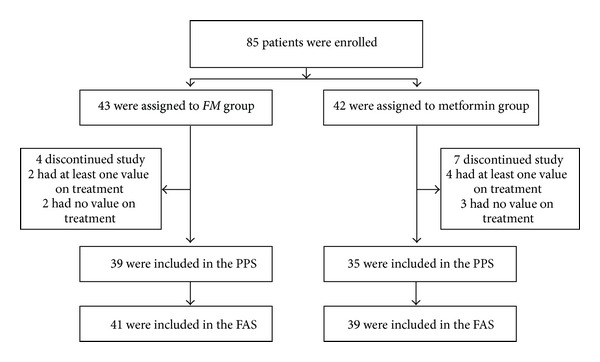


A total of 85 patients were recruited in the present study. Four patients in the *FM* group and seven patients in the Metformin group withdrew before week 12. Of these, two patients in the *FM* group and four patients in the Metformin group took at least one dose of study drug and had at least one value on treatment; therefore, the FAS was comprised of 80 subjects (41 in the *FM* group and 39 in the Metformin group) and the PPS was comprised of 74 subjects (39 in the *FM* group and 35 in the Metformin group). Enrollment, randomization and followup in the present study are depicted in Figure 1.

### 3.1. Glycemic Control

At week 12 the proportion of patients who had achieved fasting plasma glucose <7.0 mmol/L was similar in both groups (WCS: *FM* group versus Metformin group = 73% (30/41) versus 87% (34/39)). The proportion of patients who had achieved postprandial plasma glucose <10.0 mmol/L was also similar in both groups (WCS: *FM* group versus Metformin group = 68% (28/41) versus 69% (27/39)). The results of analyses for per-protocol set were in line with those for WCS (PPS analyses: for FPG, *FM* group versus Metformin group = 77% versus 86%; for 2hPG, *FM* group versus Metformin group = 72% versus 65%). 

### 3.2. Fasting Plasma Glucose and Postprandial Plasma Glucose

**Figure 2:**
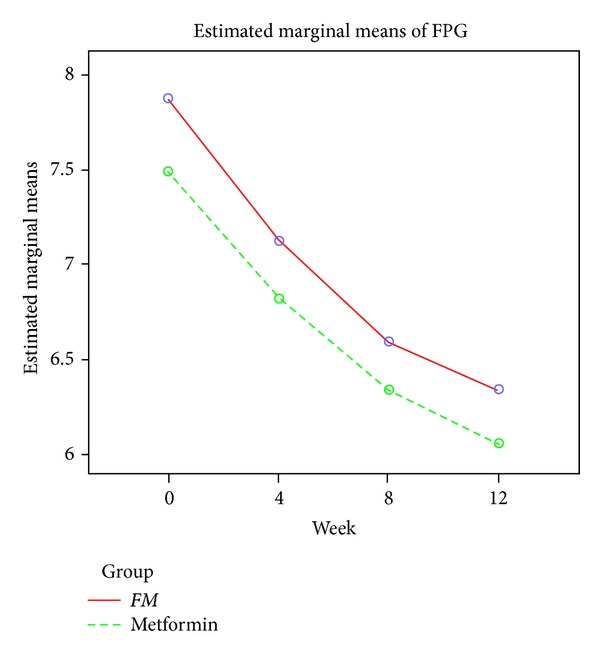


**Figure 3:**
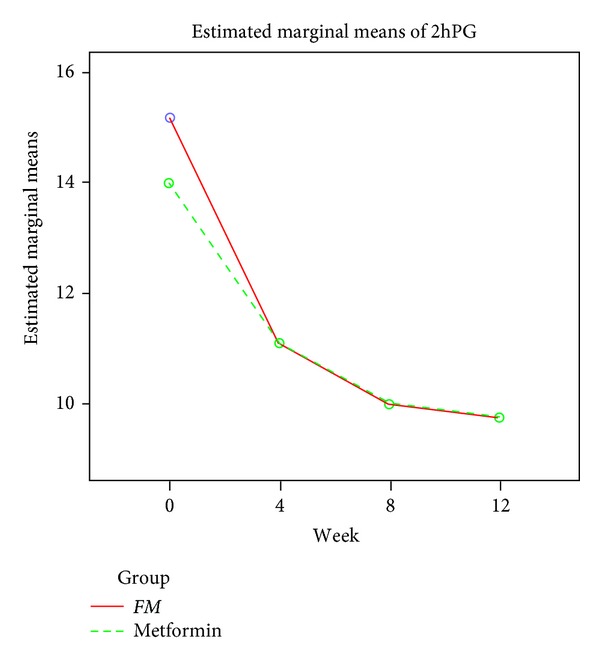


By week 12 the fasting plasma glucose concentration had decreased by 1.53 mmol/L to 6.35 mmol/L in the *FM* group and decreased by 1.44 mmol/L to 6.06 mmol/L in the Metformin group (for the comparison of *FM* with Metformin, *P* > 0.05, Table 3 & Figure 2). At week 12, the postprandial plasma glucose levels decreased by 5.45 mmol/L to 9.73 mmol/L in the *FM* group and decreased by 4.29 mmol/L to 9.72 mmol/L in the Metformin group (for the comparison of *FM* with Metformin, *P* > 0.05, Table 4 and Figure 3). The results of the perprotocol set analyses (Tables 3 and 4) were in line with those of FAS analyses.

### 3.3. Glycosylated Hemoglobin (HbA1c)

The levels of HbA1c decreased from 7.66 percent to 6.78 percent in the *FM* group and decreased from 8.23 percent to 6.76 percent in the Metformin group (*P* > 0.05). 

### 3.4. Insulin Concentrations

The fasting insulin concentrations were similar in the *FM* and Metformin groups (*P* > 0.05) and they did not significantly change during the 12-week period (data not shown). 

### 3.5. Lipid Profiles

The lipid profiles (TC, TG, LDL, and HDL) were similar in the *FM* and Metformin groups (*P* > 0.05), and they did not significantly change during the 12-week period (data not shown). 

### 3.6. Body Mass Index

For subjects whose BMI was between 23 and 25 and >25, *FM *could significantly decrease BMI, with no significant differences compared with Metformin. For subjects whose BMI <23, neither Metformin nor *FM* could decrease BMI. The changes in BMI were shown in Table 5.

### 3.7. Adverse Events

One patient in the *FM* group withdrew because of adverse events. The patient had palpitation and diarrhea, and they disappeared after the discontinuation of *FM*. The adverse events were classified as moderate in severity and probably related to *FM*. 

## 4. Discussion

Surprisingly, *Fructus Mume *formula is as effective as the most eminent hypoglycemia agent—Metformin in reducing blood glucose levels. In the WCS, there were 73 percent patients with FPG < 7.0 mmol/L. The fasting plasma glucose reduced by 1.53 mmol/L and the postprandial glucose reduced by 5.45 mmol/L in the twelve-week period. What is more, the HbA1c decreased by 0.88 percent points. The results of FAS were in line with those of PPS in significance for glycemic outcomes, which suggested that our results were robust. However, we must interpret the results cautiously because of the following reasons. 

First, blood glucose levels can be affected by many factors. The reduction in blood glucose levels cannot be attributed only to *FM*, as subjects underwent dietary and exercise therapy, and this might affect blood glucose significantly. However, since both groups received dietary and exercise therapy, the results still have clinical implications. 

Second, as the trial lasted only a short 12-week period, we were not able to arrive at any conclusions concerning the long-term effect and safety of *FM.* Regarding the safety of this formula, since there was one subject who dropped out because of palpitation and diarrhea, it raises the question of the long-term toxicity of *FM* which contains both *Rhizoma Typhonii Gigantei*, as well as *Herba Asari* (removed in this study). 

Thirdly, we went to great lengths to make the present trial well designed. However, it was impossible to carry out a double blind trial due to the obvious difference between TCM and Metformin. 

The TCM community has developed many approaches for the treatment of diabetes, both theoretically and clinically. Frankly speaking, these, may have enriched the management portfolio of diabetes, but may have impaired the standardization of TCM. Is there a common TCM way to reduce the level of blood glucose? Taking into account the *Yin-Yang* theory, we think that managing diabetes with herbs of hot or cold properties together might be a good choice. In *Yin-Yang* theory, sweetness is a flavor of the *Yang* property (an expansive process) and bitterness and sourness are flavors of the *Yin* property (contracting). One TCM explanation of diabetes is that sweet flavor develops to an extreme point (excessive *Yang*), triggering the onset. The relationship of sugar consumption is obvious here. Therefore, we use herbs of bitter flavor—such as *Rhizoma Coptidis* and herbs of sour flavor—such as *Fructus Mume* in the treatment of T2DM (bitter flavor can counteract sweet flavor because it is in direct opposition to sweetness, and sour flavor can neutralize sweet flavor). As herbs of cold property could impair the healthy Qi of the body, herbs of hot property should be added to counteract the side-effects. As *Fructus Mume *formulais such a famous classical formula, we chose it for assessing the effects of TCM in reducing blood glucose levels. In our opinion, *Fructus Mume *formula reduces the blood glucose levels by adjusting the balance of *Yin* and *Yang*. However, the question to be answered is “By removal of some of the classic formula ingredients how does it effect the balance of Yin and Yang? [sic]” Of course, it is not enough to explain the hypoglycemic effects of *FM* just by Yin-Yang theory. It is notable that although many articles declared that TCM may have insulinotropic effects, our results demonstrated that neither *FM* nor Metformin could affect the insulin concentration. Since published literature reported that *Radix Ginseng, Rhizoma Coptidis, Cortex Phellodendri,* three herbs used in *FM,* that have antioxidant effects [16, 21–23], we took the results into account and therefore hypothesized that *FM* may exert its hypoglycemic effect on type 2 diabetes primarily by improving sensitivity of peripheral tissues and hence decreasing insulin resistance and by the reducing oxidative stress.

We also should note that the baseline levels of blood glucose were not high (mean values, 7.87 mmol/L), which suggested the severity of disease of observed subjects. This indicates that the diabetes severity level, to which TCM can be applied as a monotherapy that is still limited. Regarding the differences in Western and Asian body types, the dose of Metformin (i.e., 500 mg bid) might not be strong enough to produce optimal glycemic control for Caucasians, but this dose may be appropriate as the starting therapy for Chinese patients. 

Although numerous articles declared that TCM could provide clinical benefits for the management of diabetes, most published documents were of poor methodology and no firm conclusions could be drawn. It is really very easy to yield clinical trials evaluating the effects of TCM on diabetes mellitus in some less-than-rigorous medical journals. Lack of scientific rigor is a current major problem with Chinese editors in the assimilation of scientific methodology. Some trials declared that TCM could reduce the levels of blood glucose, and some declared that TCM could provide clinical benefits for diabetic patients with microvascular or macrovascular complications—but without well-designed controlled research, it is not a solid demonstration of the evidence. 

In contrast, almost all of the systematic reviews or meta-analyses which summarized the results of published articles concluded that observational trials are generally of poor methodology and well-designed randomized controlled trials (RCT) are warranted to confirm the effects of TCM on diabetes mellitus. For example, *Liuwei Dihuang Wan* is a widely used Traditional Chinese Medicinal formula for the management of diabetes. One systematic paper examined the efficacy and safety of *Liuwei Dihuang Wan* and included five RCTs. Although the five observational RCTs were of poor methodology, this review still reached the conclusion that *Liuwei Dihuang Wan* is effective and safe for type 2 diabetes [24] (which really baffles readers). Obviously, to date, most clinical evidence from the TCM community is not persuasive. Our study, albeit not conclusive, introduced higher methodology in TCM clinical trials. At any rate, the present pilot study deserves a seat in the field of TCM for diabetes because it introduces the strict methodology and it is, without doubt, brave enough to evaluate the effect of TCM, when used as a monotherapy, on “hard outcomes.”

Compared with the established hypoglycemic effects of western medicine, the blood glucose-lowering effect of TCM remains controversial. However, it is a topic many experts would like to clarify. He et al. [25] evaluated the antidiabetic efficacy and mechanisms of 34 TCMs. Their results showed that 13 out of the 34 herbs showed a statistically significant plasma glucose-lowering action compared with the diabetic control group. 

To our current knowledge, most TCM formulae aimed at diabetes focus on alleviating diabetic symptoms other than decreasing blood glucose. Although few TCM formulae have recognized blood glucose-lowering effects, it is not difficult to yield articles in medical journals where the authors declared their test TCM had blood glucose-lowering effects. A brief look at the evidence demonstrates that TCM may have hypoglycemic potential. In a Chinese article, Zhong et al. [26] concluded “the TCM *Yitangyin* (YTY) granule is an effective hypoglycemic agent.” Zhao and coworkers [27] reported that a TCM formula, referred to as *JCU*, has sustained glucose-lowering effects in male Zucker diabetic fatty rats. Li et al. [28] assessed the effect of *qiangyi jiangtang* capsules (*QJC*) on diabetes mellitus model Wistar rats, and their results demonstrated “*QJC* could remarkably lower the levels of blood glucose, HbA1c.” Since published literature with respect to the hypoglycemic effects of TCM is increasing, the hypoglycemic effect of TCM remains an explorative issue and the debate about it needs to be clarified.

## 5. Conclusions and Looking Forward

In summary, *Fructus Mume* formula may reduce the levels of blood glucose in patients with type 2 diabetes to some extent. The present study also discloses the potential of TCM strategy with the use of herbs of cold or hot property together to adjust the balance of *Yin* and *Yang* in the treatment of type 2 diabetes. Future research on this topic should address (1) *FM* ingredients which are toxic, (2) a closer look at individual patient characteristics that may be prone to the side effects, and (3) if TCM methods are able to significantly reduce the levels of blood glucose across levels of BMI.

## Figures and Tables

**Table 1:** Traditional chinese medicinal herbs used in the *Fructus Mume* formula.

PinYin name	Latin name	English name	Original dose	Modification
Wumei	*Fructus Mume *	Dark plum fruit	300 pieces	30 g
Xixin	*Herba Asari *	Manchurian wildginger	6 tael	Removed
Ganjiang	*Rhizoma Zingiberis *	Dried ginger	10 tael	15 g
Huanglian	*Rhizoma Coptidis *	Golden thread	16 tael	30 g
Danggui	*Radix Angelicae Sinensis *	Chinese angelica	4 tael	10 g
Fuzi	*Rhizoma Typhonii Gigantei *	Giant typhonium rhizome	6 tael	20 g
Huajiao	*Fructus Zanthoxyli *	Prickly ash peel	4 tael	5 g
Guizhi	*Ramulus Cinnamomi *	Cassia twig	6 tael	10 g
Renshen	*Radix Ginseng *	Ginseng	6 tael	10 g
Huangbai	*Cortex Phellodendri *	Amur corktree bark	6 tael	20 g

**Table 2:** Demographic and baseline characteristics of the FAS population of the two groups of patients.

Characteristic	*FM* group	Metformin group	*P* value
Sex			
male	25	14	0.025
female	16	25	
Age (years)	54.37 ± 8.89	54.77 ± 11.83	0.864
Diabetes duration (months)	16.41 ± 21.15	14.34 ± 27.20	0.703
Body weight (Kg)	64.76 ± 11.85	60.79 ± 7.39	0.085
BMI (Kg/m^2^)	24.31 ± 4.34	24.04 ± 2.85	0.757
SBP (mmHg)	118.59 ± 11.02	121.31 ± 13.12	0.335
DBP (mmHg)	79.85 ± 7.42	78.57 ± 8.02	0.48
TC (mmol/L)	4.90 ± 1.01	4.59 ± 1.33	0.286
TG (mmol/L)	2.53 ± 3.48	2.37 ± 2.48	0.832
HDL (*μ*mol/L)	1.68 ± 0.54	1.57 ± 1.20	0.630
LDL (*μ*mol/L)	3.03 ± 0.94	3.06 ± 0.91	0.898
HbA1c (%)	7.66 ± 1.11	8.23 ± 1.95	0.352
FPG (mmol/L)	7.871 ± 1.461	7.494 ± 1.544	0.266
2hPG (mmol/L)	15.182 ± 2.715	14.006 ± 3.061	0.073
Insulin concentration (mU/L)	8.65 ± 5.60	6.89 ± 3.90	0.416

**Table 3:** Changes in FPG (means ± SD, mmol/L).

	Week 0	Week 4	Week 8	Week 12
PPS				
*FM* group	7.934 ± 1.470	7.164 ± 1.176	6.609 ± 1.117	6.339 ± 1.308
Metformin group	7.524 ± 1.577	6.831 ± 1.138	6.304 ± 1.009	5.966 ± 1.000
FAS				
*FM* group	7.871 ± 1.461	7.129 ± 1.161	6.601 ± 1.094	6.345 ± 1.279
Metformin group	7.494 ± 1.544	6.823 ± 1.129	6.349 ± 1.014	6.056 ± 1.029

**Table 4:** Changes in 2hPG (means ± SD, mmol/L).

	Week 0	Week 4	Week 8	Week 12
PPS				
*FM* group	15.274 ± 2.748	11.045 ± 2.56	9.901 ± 1.844	9.646 ± 2.691
Metformin group	14.076 ± 3.178	11.11 ± 2.236	9.958 ± 1.790	9.633 ± 1.890
FAS				
*FM* group	15.182 ± 2.715	11.063 ± 2.544	9.974 ± 1.891	9.732 ± 2.696
Metformin group	14.006 ± 3.061	11.085 ± 2.148	10.000 ± 1.702	9.716 ± 1.803

**Table 5:** Changes in BMI (means ± SD, Kg/m^2^).

		Week 0			Week 12	
	BMI < 23	23 < BMI < 25	BM I > 25	BMI < 23	23 < BMI < 25	BMI > 25
*FM* group	20.07 ± 1.23	24.07 ± 0.61	28.43 ± 3.64	20 ± 1.15	23.49 ± 0.75*	27.35 ± 3.57*
Metformin group	20.77 ± 1.18	24.08 ± 0.53	26.64 ± 1.47	20.49 ± 0.95	23.76 ± 0.65*	25.68 ± 1.82*

*Indicates that there is significant difference between the values at week 0 and week 12 (*P* < 0.05).
